# Subjective age of acquisition norms for 30,849 Russian words

**DOI:** 10.3758/s13428-026-03138-2

**Published:** 2026-08-31

**Authors:** Andrei A. Grigoriev, Konstantin V. Sugonyaev, Svetlana A. Pashneva, Ekaterina A. Valueva, Nikolai A. Grigorev

**Affiliations:** 1 Institute of Psychology, Russian Academy of Sciences, 13-1 Yaroslavskaya Str., Moscow, 129366 Russian Federation; 2 Department of Foreign Languages, Kursk State University, Radishcheva Str., 33, 305000 Kursk, Russian Federation; 3 Faculty of Archaeology, University of Warsaw, Krakowskie Przedmieście Str., 26/28, 00-927 Warsaw, Poland

**Keywords:** Age of acquisition, Lexical norms, Russian language, Psycholinguistic databases, Vocabulary development, Lexical processing

## Abstract

Age of acquisition (AoA) is a key psycholinguistic variable known to influence lexical and conceptual processing across a broad range of tasks. However, existing Russian datasets are small and mainly limited to picturable nouns and verbs. In this study, we collected AoA ratings for 30,849 Russian words that represent a broad vocabulary range. Ratings were collected from 2,201 adult respondents in both in-person and online sessions. Reliability assessed via bootstrap split-half correlations was high (mean *r* = 0.895, 95% CI [.893, .897]). To validate the subjective AoA estimates, we administered vocabulary tests to school students from grades 2, 4, 6, 8, and 10. Two validation procedures were used: grade-level validation and within-grade validation. The former related the grade level at which an item was reliably known with the adult ratings, the latter related adult AoA to students’ accuracy. Subjective AoA strongly predicted objective AoA measures (*r* = 0.805) and showed consistent negative relations with student accuracy across grades. Correlations with existing Russian AoA datasets (*r* = 0.68–0.91) further supported the reliability of the norms. The new ratings also showed expected correlations with word frequency, length, concreteness, imageability, and familiarity. The resulting norms provide the largest and most comprehensive AoA resource for the Russian language and can support future research in psycholinguistics, lexical processing, and language development. All norms are publicly available on OSF.

## Introduction

Age of acquisition (AoA), that is, the age at which L1 words are learned, is an important factor in cognitive processing. Its effect has been demonstrated in a variety of tasks. For example, pictures of objects whose names are acquired earlier in life are named faster (e.g., Carroll & White, 1973; Ellis & Morrison, 1998; Catling et al., 2008), and words acquired earlier are read faster (e.g., Brysbaert et al., 2000a; Butler & Hains, 1979; Morrison & Ellis, 1995). AoA affects both response times (e.g., Brysbaert et al., 2000a; Butler & Hains, 1979; Morrison & Ellis, 1995) and error rates (Brysbaert et al., 2000a) in the lexical decision task. It also affects response times and the average number of different associates produced for stimulus words in the discrete word–associate generation task (Brysbaert et al., 2000b). The AoA effect has also been observed in a semantic word categorization task (Brysbaert et al., 2000b), but not in picture categorization (Morrison et al., 1992; Holmes & Ellis, 2006). It is therefore not surprising that considerable research effort has been devoted to collecting normative AoA data.

Researchers have collected these data mainly by asking adult participants to estimate and report the age at which they learned specific words. The values obtained in this way are called subjective AoA ratings. Subjective AoA ratings, typically collected alongside other psycholinguistic variables, are available for Chinese (Liu et al., 2011), Croatian (Peti-Stantić et al., 2021), Dutch (Brysbaert et al., 2014a; Verheyen et al., 2020), English (Gilhooly & Logie, 1980; Kuperman et al., 2012; Stadthagen-Gonzales & Davis, 2006), French (Bonin et al., 2022; Ferrand et al., 2008), German (Schröder et al., 2012), Icelandic (Pind et al., 2000), Italian (Montefinese et al., 2019), Japanese (Mochizuki & Ota, 2025), Portuguese (Cameirão & Vicente, 2010), Russian (Akinina et al., 2015; Kolbeneva & Alexandrov, 2010; Miklashevsky, 2018; Pashneva, 2014; Tsaparina et al., 2011), Spanish (Alonso et al., 2015), Turkish (Göz et al., 2017), and many other languages (Łuniewska et al., 2016; Łuniewska et al., 2019).

In many studies in which AoA ratings were collected, some type of rating scale was used. For example, Gilhooly and Logie (1980) used a 7-point scale (1 = 0–2 years, 7 = 13 years and older), whereas Akinina et al. (2015) used a 5-point scale (1 = 0–3 years, 5 = 12 years and older). Other researchers (Kuperman et al., 2012; Montefinese et al., 2019; Łuniewska et al., 2016; Łuniewska et al., 2019), however, prefer to ask respondents to report the AoA of words in years. The latter method of collecting AoA data appears preferable. As Kuperman et al. (2012) note, the use of rating scales “artificially restricts the response range and is also more difficult for participants to use” (p. 980).

AoA ratings collected for different languages correlate with each other. Łuniewska et al. (2016) reported reliability-adjusted Spearman correlations between AoA ratings for 25 languages. The lowest correlation (.62) was found between Hungarian and Greek, and the highest (.96) between Polish and Slovak. In a subsequent study, Łuniewska et al. (2019) added data for seven new languages. The reliability-adjusted Spearman correlations among these seven languages ranged from.45 to.85, whereas the correlations between the 25 languages from the earlier study and the seven new languages ranged from.46 to.94.

Some studies have also examined the relationships between respondents’ AoA ratings and their demographic characteristics. According to the results of Kuperman et al. (2012), the mean AoA rating reported by women is slightly higher than that reported by men. Łuniewska et al. (2016), however, did not find a significant difference between men and women matched for age, education level, and first language. In the study by Kuperman et al. (2012), AoA ratings showed a weak positive correlation with participants’ age. In the study by Łuniewska et al. (2016), the correlation between average AoA ratings and participants’ age was not significant. Interestingly, both Kuperman et al. (2012) and Łuniewska et al. (2016) found no relationship between AoA ratings and respondents’ education level.

Far fewer studies have focused on collecting objective AoA values, likely because the data collection procedures are much more complex and time-consuming. In several studies, such values were obtained by presenting children of different ages with pictures and asking them to name the depicted objects (e.g., Grigoriev & Oshhepkov, 2013; Morrison et al., 1997). In addition, AoA norms for a large set of English words were derived from data obtained by testing students across school grade levels and college cohorts (Brysbaert & Biemiller, 2017).

The relationships of both AoA ratings and objective AoA values with other psycholinguistic variables (e.g., word length, word frequency, imageability) are well documented. The correlation coefficients obtained for different languages are often quite similar. For example, the Spearman correlations between AoA ratings and word length reported in Łuniewska et al. (2019) for seven languages range from.14 to.50, whereas the correlations with word frequency reported for four languages range from −.39 to −.56. The correlations between objective AoA values and word imageability and object familiarity reported by Morrison et al. (1997) for English are −.549 and −.439, respectively, whereas for Russian these correlations are −.430 and −.278.

The purpose of the present study is to collect AoA ratings covering a broad range of Russian vocabulary and to validate them. All existing Russian AoA datasets are limited to relatively small word samples, typically consisting of only a few hundred items. Thus, Tsaparina et al. (2011) obtained AoA ratings for 260 nouns or phrases (e.g., зyбнaя щeткa = toothbrush) denoting depicted objects. Pashneva (2014) collected AoA ratings for 190 verbs, and Akinina et al. (2015) obtained ratings for 375 verbs. In addition, AoA ratings were obtained for 696 nouns (Akinina et al., 2014) and for 414 verbs (Akinina et al., 2016). Kolbeneva and Alexandrov (2010) collected AoA ratings for 475 adjectives. Miklashevsky (2018) obtained AoA ratings for 506 nouns, both concrete and abstract. Objective AoA values for 375 nouns or phrases were reported by Grigoriev and Oshhepkov (2013). Subjective AoA ratings for Russian words or phrases collected in these studies were generally not validated, except for the correlation between the ratings reported by Tsaparina et al. and the objective values reported by Grigoriev and Oshhepkov (.673), as noted in Grigoriev and Oshhepkov (2013).

In the present study, AoA ratings were collected for 30,849 Russian words covering a broad range of Russian vocabulary. The ratings were provided by adult native speakers recruited both in person and online. We validated the ratings in school samples using specially designed verbal tests administered across multiple grade levels. The validation followed two complementary procedures. For grade-level validation, we derived an objective AoA measure from the grade level at which an item was reliably known and correlated it with the adult ratings. For within-grade validation, we examined the relationship between adult AoA ratings and students’ accuracy on matched item sets.

## Method

### Participants

About 3,000 participants took part in the collection of subjective AoA ratings. The in-person respondents were primarily first-year students and faculty members from various universities. For the online data collection, participants were recruited through the Yandex.Toloka platform and received 200 RUB (approximately $2.50) as compensation. After the application of several exclusion criteria (see the Results section), 2,201 protocols (approximately 73%) were retained for the final analysis. Of these, three did not include information on the respondent’s gender. Among the 2,198 protocols with reported gender information, 1,251 were completed by males and 947 by females. One protocol did not include the respondent’s age. Based on the remaining 2,200 protocols, the mean age of the respondents was 27.2 years (*SD* = 11.0).

### Stimuli

We collected subjective AoA ratings for 30,900 Russian words organized into 103 word lists. Each list consisted of 300 target words, 10 calibrator words, and 30 control words whose ratings had been previously established. The calibrator words were used (following Kuperman et al, 2012) to show respondents the possible range of ratings, whereas the control words were used to check the validity of their responses. The compilation of the word lists, as well as the data collection, proceeded in three phases. In 2022, 25 word lists were compiled; in 2023, 55 word lists were compiled; and in 2024, 23 word lists were compiled. Some words included in the lists in 2022 and 2023 were mistakenly reused in subsequent phases. For this reason, the 103 word lists contained 30,849 unique target words.

The vast majority of the target words were drawn from the frequency dictionary by Lyashevskaya and Sharov (2015), which contains 49,702 words with a frequency of 0.4 instances per million (IPM) or higher. Many of these words belong to the same word family, for example, *тигp* (tiger), *тигpицa* (tigress), and *тигpeнoк* (tiger cub). As a rule, although not always, we included only one member of each such family in the stimulus set. We also excluded ethnonyms and related forms, such as *фpaнцyз* (Frenchman), *фpaнцyжeнкa* (Frenchwoman), and *фpaнцyзcкий* (French), as well as colloquial forms, such as *вeлик* for *вeлocипeд* (bike), and some other classes of words listed in the dictionary. A number of words not included in this dictionary were also added to the stimulus set. These were words presented in Russian category norms (Grigoriev & Oshhepkov, 2005) and therefore known to Russian speakers, as well as some other words that were not frequent enough to be included in the frequency dictionary but seemed familiar to modern Russian speakers. As a rule, words were presented in their lemmatized form. In some cases, however, nouns were presented in the plural when that form is more common in actual usage.

Among the target words in each list, 134 were nouns, 76 verbs, 70 adjectives, 17 adverbs, and 3 belonged to other parts of speech. This distribution corresponded to the distribution of parts of speech in the dictionary by Lyashevskaya and Sharov (2015). As a rule, only one word from a given semantic category (e.g., birds, colors) was included in a given list. When compiling the lists, we aimed to avoid, or at least minimize, cases in which words from the same derivational family (e.g., *xитpый* “cunning” and *xитpocть* “craftiness”) appeared in the same list.

**Fig. 1:**
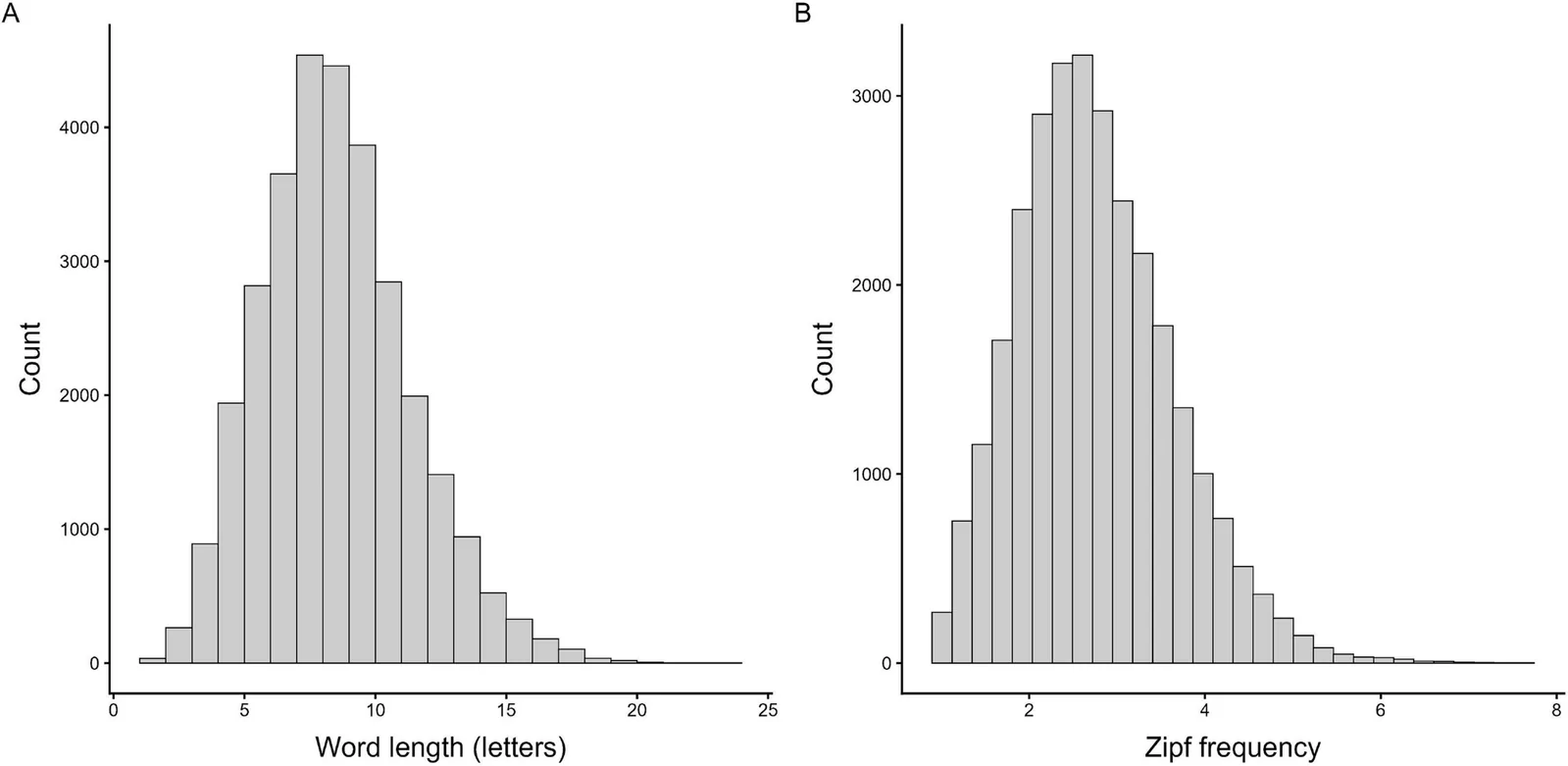
Distribution of word lengths (**A**) and Zipf frequencies (**B**) in the stimulus set

Word length in the final stimulus set ranged from 1 to 24 letters (*M* = 9.09, *Mdn* = 9.00, *SD* = 2.85). Word frequency, indexed using Zipf frequency values from *wordfreq* (Speer, 2022), ranged from 1.01 to 7.63 (*M* = 2.76, *Mdn* = 2.66, *SD* = 0.88). Figure 1 presents the distributions of word length and Zipf frequency.

### Data collection

The data collection procedure broadly followed the methodology used by Kuperman et al. (2012) for collecting AoA ratings and by Brysbaert et al. (2014b) for collecting concreteness ratings. Respondents first read instructions explaining the purpose of the study, the definition of “word acquisition,” and the rating procedure. They were asked to estimate the approximate age, in years, at which they believed they had learned each word. If a word was unfamiliar to them, they were asked to indicate this. The instructions also noted that the beginning of each word list contained 10 calibrator words, which were intended to help respondents better understand the procedure and the possible range of ratings. Respondents were asked to provide the most accurate estimates possible, but were also cautioned not to spend too much time on any single word.

Data were collected both in in-person paper-and-pencil sessions (approximately 58% of the protocols) and online. The online data collection was implemented using the PsyToolkit platform (Stoet, 2010, 2017). Data collection proceeded in three phases. In 2022, ratings were obtained for 7,500 words; in 2023, for 16,500 words; and in 2024, for 6,900 words. The sets of calibrator words and control words were changed over the course of data collection. The instructions for the paper-and-pencil format and the lists of calibrator words are provided in the Appendix.

## Results

### Data preparation

The data for words that appeared in more than one list were merged. Data trimming was performed as follows.

1. Each protocol was checked for autocorrelation (lag = 1). As a rule, protocols were excluded if the value exceeded 0.3.
2. Each protocol was checked for its correlation with the previously established ratings for the control words. Protocols were typically excluded if this correlation was below 0.7.
3. The respondent’s mean AoA rating for the target words in the presented list was compared with the overall mean rating for the same list across all respondents. If the respondent’s mean deviated from the overall mean by more than two standard deviations, the protocol was subject to exclusion.
4. Protocols were excluded if the minimum AoA rating exceeded 4.
5. Protocols with more than 60 “don’t know” responses were excluded.
6. Each protocol was checked for the correlation between the respondent’s ratings and the mean ratings for the corresponding word list. The cutoff value was 0.7.

In certain cases, a small deviation from one of the cutoff values was allowed if the respondent’s data met the other quality criteria. In addition, during online data collection, the response time for each word was recorded. On this basis, individual word responses, rather than entire protocols, were excluded, namely, responses with latencies shorter than 800 ms. In addition to applying the criteria listed above, all responses were also inspected manually. If a respondent gave a large number of implausible ratings (e.g., very low AoA ratings for complex words or very high ratings for simple words), the entire protocol could also be rejected.

Out of 3,025 protocols, 824 were excluded. Among the remaining 2,201 protocols, there were occasional missing responses or responses that did not follow the instructions. In the case of online data collection, some responses were also missing because of technical issues. As a result of missing data, excluded responses, and technical issues, only 19 valid responses were obtained for 35 target words, with a valid response defined as either an AoA rating or a “don’t know” response. For all remaining words, 20 or more valid responses were collected.

### Reliability of subjective AoA ratings

Reliability was assessed using a bootstrap split-half analysis. For each word, respondents were randomly divided into two equal or nearly equal subsamples, mean AoA ratings were calculated for each subsample, and the correlation between subsamples was computed. Across 1,000 iterations, the mean raw split-half correlation was *r* =.894, 95% CI [.893,.897], and the mean Spearman–Brown-corrected split-half reliability was *r* =.944, 95% CI [.943,.945], indicating high reliability of the AoA ratings.

### Validity of subjective AoA ratings

To validate the subjective AoA ratings obtained in the present study, we conducted testing using specially developed verbal tests. Each test item required participants to select (from five options) the word or phrase that best matched the target word in meaning. When constructing the test items, we were guided by the following principles: (1) the target word should have either a single meaning or, if it has multiple meanings, one clearly dominant meaning; (2) the correct answer could be a synonym, hyponym, or hypernym of the target word, or a relatively short and easily understandable phrase that most accurately conveyed its meaning and was suitable as the correct answer; (3) the correct answer should be simpler than the target word; (4) situations in which the target word and the correct answer begin with the same letter or share a root should be avoided; if this was unavoidable, then one or two distractors should also begin with the same letter, whereas the remaining distractors should begin with different letters; (5) the answer options should have similar grammatical forms or, if this was not possible, should be divided into two groups with similar grammatical forms within each group; (6) the answer options should be equally distant from each other semantically, or should be grouped into two semantic clusters; and (7) if the target word was semantically complex, one of the distractors should be of comparable complexity, to reduce the likelihood of correct guessing.

Two distinct validation procedures were used: grade-level validation and within-grade validation.

**Grade-level validation.** This procedure used a 300-item test administered in grades 2, 4, 6, 8, and 10. While students in grades 6–10 completed the full test, students in grades 2–4 received only those items that had previously been correctly answered by most older students. The set of items for second-grade students was further divided into two parts, each administered to a different subgroup of participants. The test included a “don’t know” response option.

Grade-level validation estimated objective AoA as the school grade at which each word was known by students and then correlated these estimates with subjective AoA ratings. A word was considered known by students in a particular grade if at least 75% of them correctly answered the corresponding test item and students in all higher grades also answered it correctly; this latter condition did not apply to Grade 10. The AoA of a word was estimated on the basis of grade level: words known by second-grade students received a score of 2, those known by fourth-grade students a score of 4, and so on. If a word was not known by tenth-grade students, it received a score of 12.

The test was administered in schools in the Moscow region and the city of Kursk (total *N* = 145). Detailed demographic information is provided in Table 1.

**Table 1:** Demographic characteristics for student participants in the grade-level validation

Grade	*N*	Boys	Girls	Age not specified	Age range (years)
2nd	47	21	26	2	8–9
4th	24	10	14	0	10–11
6th	27	11	16	1	12–13
8th	21	12	9	0	13–15
10th	26	13	13	3	16–17

The Pearson correlation between these objective AoA estimates and the subjective AoA ratings was.805, indicating strong correspondence between the two measures.

**Within-grade validation.** The second validation procedure was conducted twice, with a slight modification of the test materials between the two rounds. The test items did not include a “don’t know” response option. In the first round, 720 test items were used and grouped into four test sets: 300 items for Grade 8, 300 for Grade 6, 240 for Grade 4, and 160 for Grade 2. The subjective AoA of the words included in each set approximately corresponded to the typical age of students in the respective grade (see Sugonyaev & Grigoriev, 2023, for details). Each test was divided into two subtests in which the test words were balanced in terms of both the mean and the range of subjective AoA. Each participant completed one of the two subtests. Within each subtest, the words were always presented in ascending order of subjective AoA ratings.

In the second round, conducted 8–10 months after the first one with different groups of students, a small number of test items were modified and the test sets were partially restructured (for details, see Sugonyaev & Pashneva, 2024). Because data collection took place in the middle of the academic year rather than at the end, as in the first round, the students were generally younger. Therefore, 20 items containing the latest-acquired words were excluded. As a result, 700 test items were used: 300 in the Grade 8 test set, 280 in the Grade 6 test set, 240 in the Grade 4 test set, and 160 in the Grade 2 test set. At the teachers’ request, the number of items in the Grade 6 subtests was reduced from 150 to 140.

The tests were administered in schools in the Moscow region and the city of Kursk (total *N* = 700). Detailed demographic information by grade and round is provided in Table 2. As noted earlier, Round 1 was conducted at the end of the school year, which resulted in participants being older than those in Round 2, conducted in the middle of the school year.

**Table 2:** Demographic characteristics for student participants in within-grade validation

Grade	Validation round	Boys	Girls	Total	Mean age	*SD*
2	1	46	47	93	8.96	0.34
2	2	49	59	108	8.38	0.33
4	1	47	47	93	10.90	0.34
4	2	52	69	121	10.33	0.31
6	1	24	30	54	12.90	0.37
6	2	28	40	68	12.67	0.31
8	1	30	42	72	14.80	0.34
8	2	48	43	91	14.57	0.36

For each grade level, correlations between the subjective AoA ratings of the words and the percentages of correct responses to the corresponding test items were calculated separately for each subtest for Rounds 1 and 2 and then averaged. Table 3 presents the mean correlations for each grade level. Both the observed correlations (*r*) and the correlations corrected for measurement error in the test scores and for range restriction in the subjective AoA ratings of the words included in each grade-level test (*R*) are reported. The latter correction was performed using Thorndike’s Case II formula (Thorndike, 1949).

$$R=\frac{{Ur}_{c}}{\sqrt{{U}^{2{r}_{c}^{2}-{r}_{c}^{2}+1}}}$$

where *U* is the ratio of unrestricted to restricted standard deviations for *x, R* is the unrestricted validity coefficient, and *r*_c_ is the range-restricted (observed) validity coefficient corrected for measurement unreliability.

**Table 3:** Observed (*r*) and corrected (*R*) correlations between the proportion of correct responses to test items and subjective AoA scores

Grade level	*r*	*R*
2	−.416	−.769
4	−.485	−.858
6	−.426	−.831
8	−.474	−.780

All correlations in Table 3 are negative, i.e., the higher the AoA rating of a word, the lower the proportion of correct responses to the corresponding test item. This pattern supports the validity of our AoA ratings.

Additionally, the validity of the subjective AoA ratings was assessed by comparing them with the objective AoA values reported by Grigoriev and Oshhepkov (2013), who asked children of different ages to name objects depicted in pictures. The correlation between the subjective and objective AoA values was.698. Thus, these results provide further evidence for the validity of the subjective AoA estimates obtained in the present study.

### Correlations with available Russian subjective AoA norms

Several Russian datasets containing subjective AoA ratings are currently available (Akinina et al., 2014; Akinina et al., 2015; Akinina et al., 2016; Kolbeneva & Alexandrov, 2010,1; Miklashevsky, 2018; Pashneva, 2014; Tsaparina et al., 2011). In the datasets reported by Akinina et al. (2014, 2015, 2016) and Tsaparina et al. (2011), AoA was rated on a 5-point scale, where 1 indicated that a word was acquired between 0 and 3 years of age and 5 indicated that it was acquired at 12 years of age or later. In the norms reported by Kolbeneva and Alexandrov (2010), AoA was rated on a 7-point scale, where 1 indicated acquisition between 0 and 2 years of age and 7 indicated acquisition at 13 years of age or later. In contrast, in the datasets reported by Miklashevsky (2018) and Pashneva (2014), AoA was estimated in years.

The correlation with the data reported by Tsaparina et al. (2011) was.905 (*N* = 225). The corresponding correlations were.853 (*N* = 190) for Pashneva (2014),.911 (*N* = 299) for Kolbeneva and Alexandrov (2010), and.905 (*N* = 494) for Miklashevsky (2018). The correlation with the data reported by Akinina et al. (2014, 2015, 2016) was.882 (*N* = 654) for nouns,.683 (*N* = 352) for verbs, and.822 (*N* = 1004) for all words. Thus, comparisons with subjective AoA scores obtained in other studies support the reliability of our ratings.

### Relationships between subjective AoA ratings and other psycholinguistic variables

We examined the correlations between the obtained AoA ratings and several other psycholinguistic variables, namely word length, frequency, concreteness, imageability, and familiarity. Word frequency was taken from two sources. First, we used the frequency dictionary by Lyashevskaya and Sharov (2015), which reports IPM values based on the Russian National Corpus. Second, we used Zipf frequency scores computed with the wordfreq Python library (version 3.1.1; Speer, 2022), which is based on the Exquisite Corpus.^2^ Concreteness ratings were taken from Grigoriev and Pashneva (2022) and from the supplementary materials to Solovyev et al. (2022). The latter source provides two datasets of concreteness ratings, one for 1,000 words and one for 500 words. We did not merge these datasets. In the 1,000-word dataset, three words, *бaзa* (base), *знaниe* (knowledge), and *тoннa* (ton), were listed twice with different ratings; in the 500-word dataset, this was the case for one word, *yдap* (a hit). For these items, we used the average of the two ratings. Imageability ratings were taken from Grigoriev et al. (2009), Tsaparina et al. (2011), and Pashneva (2014). Familiarity ratings were obtained from Tsaparina et al. (2011), Grigoriev et al. (2010), and Pashneva (2014).

The correlation between AoA and word length was *r* =.282 when length was measured in letters and *r* =.293 when it was measured in syllables. The correlation between AoA and the log-transformed IPM values from the frequency dictionary by Lyashevskaya and Sharov was −.451, whereas the correlation between AoA and the Zipf frequency scores based on the Exquisite Corpus was −.380. Correlations between AoA ratings and ratings of concreteness, imageability, and familiarity are presented in Table 4.

**Table 4:** Correlations between subjective AoA ratings and ratings of concreteness, imageability, and familiarity (*N* in parentheses)

Study	Concreteness	Imageability	Familiarity
Grigoriev & Pashneva, 2022	−.382 (1135)*		
Soloviev et al, 2022, 1,000 words	.380 (979)*		
Soloviev et al, 2022, 500 words	.357 (475)*		
Grigoriev et al., 2009		−.571 (442)**	
Miklashevsky, 2018		−.490 (494)**	
Tsaparina et al., 2011		−.563 (225)**	−.503 (225)
Grigoriev et al., 2010			−.386 (266)
Pashneva, 2014		−.531 (190)**	−.524 (190)
Akinina et al., 2014; Akinina et al., 2016 nouns		.505 (654)**	
Akinina et al., 2015 verbs		.328 (352)**	
Akinina et al., 2014; Akinina et al., 2016; Akinina et al., 2015		.416 (1004)**	

* In Soloviev et al. study 1 = concrete and 5 = abstract, while in Grigoriev & Pashneva study 7 = concrete and 1 = abstract

** In Akinina et al. studies 1 = easy to imagine and 5 = hard to imagine, while in other studies 7 (or 5) = easy to imagine and 1 = hard to imagine

Taken together, the results show clear patterns. Words acquired later tend to be longer and less frequent, as well as less concrete, less imageable, and less familiar. Most effects are of moderate size (*r* about.33–.57), with the strongest associations observed for imageability. The consistency of the results across two frequency sources and multiple normative datasets supports the convergent validity of the AoA measure, whereas differences in effect sizes across studies likely reflect variation in item sets and rating procedures.

### Relationships between respondents’ age, mean AoA estimates, and the number of “don’t know” responses

Respondents varied widely in age, which allowed us to examine how age was related to their ratings and “don’t know” responses. Age showed a weak positive correlation with the mean AoA rating (*r* =.119, *p* <.001) and a negative correlation with the number of “don’t know” responses (*r* = −.370, *p* <.001). Thus, older respondents gave later AoA estimates and produced fewer “don’t know” responses than younger respondents.

## Discussion

The present study introduces the largest and most comprehensive set of subjective AoA ratings available for the Russian language to date. By collecting ratings for 30,849 words from a large and heterogeneous adult sample, we substantially expand the lexical coverage of existing Russian norms, which have so far been mainly restricted to picturable nouns and verbs and typically included only a few hundred items.

A first important outcome of the study is the high reliability of the obtained ratings. The bootstrap split-half correlation (.895) demonstrates that adult respondents provide stable and consistent AoA estimates even for a very large set of items. This level of reliability is comparable to or higher than what has been reported for AoA norms in other languages. Moreover, the correlations with previously published Russian AoA datasets (ranging from.68 to.91 depending on part of speech and source) provide additional evidence of reliability. These substantial associations indicate that despite methodological and lexical differences across studies, subjective AoA remains a robust construct that can be measured consistently.

A second major contribution is the validation of subjective AoA scores using two independent vocabulary-testing procedures. Grade-level validation revealed a strong correspondence between subjective AoA and the grade at which schoolchildren reliably understood target words (*r* =.805). This result indicates that adults’ retrospective judgments closely track the developmental trajectory of word meaning acquisition. The within-grade validation further supported this conclusion by showing robust negative correlations between subjective AoA and accuracy within each grade. After correcting for measurement error and range restriction, these correlations increased substantially, suggesting that subjective AoA captures meaningful variation in children’s actual lexical knowledge across different ability levels. Together, these findings provide converging evidence that the subjective AoA ratings offer a valid approximation of the objective acquisition timeline.

Correlations with other psycholinguistic variables also showed patterns consistent with prior research. Reported correlations between AoA and different measures of word length vary considerably, from.057 (Lotto et al., 2010) to.56 (Cameirao & Vicente, 2010), with many values falling in the.2–.4 range. Our findings (*r* =.28) fall within this interval. Our correlations with word frequency measures (−.380 and −.451) likewise fall within the range of published findings, from −.34 (Cameirão & Vicente, 2010) to −.75 (Ferrand et al., 2008). The correlations with concreteness (in absolute value, from.357 to.382) also lie within the range reported in previous studies, where the corresponding values range from.31 (Verheyen et al., 2020) to.61 (Cameirão & Vicente, 2010). The same applies to correlations with imageability (from −.328 to −.571), where reported values range from −.317 (Göz et al., 2017) to −.738 (Bonin et al., 2022). Thus, as expected, earlier acquired words tended to be shorter, more frequent, more concrete, more imageable, and more familiar.

The study also offers insights into respondent-level factors influencing AoA judgments. Older respondents tended to give slightly later AoA estimates and to mark fewer words as unknown. The former result is consistent with the findings of Kuperman et al. (2012), who reported a correlation of.07 between AoA ratings and respondents’ age, whereas the latter is not. In their study, no relationship was found between a similar measure, namely the ratio of numerical responses to total responses, and demographic variables. Perhaps the difference in the time period of data collection contributed to this discrepancy: our data were collected in 2022–2024, whereas the norms reported by Kuperman et al. were published in 2012. In any case, examining the temporal dynamics of vocabulary knowledge in different populations is an important task for future research.

Beyond methodological advances, the present norms have clear practical value. Many psycholinguistic studies rely on AoA as a predictor of lexical processing speed, error rates, and semantic activation patterns. Until now, the lack of a large-scale AoA resource for Russian has hindered the development of experimental materials and has limited cross-linguistic comparisons. The new dataset provides researchers with a tool comparable in scope to AoA norms available for English (Kuperman et al., 2012), Dutch (Brysbaert et al., 2014a), Spanish (Alonso et al., 2015), and contributes to the effort to map lexical development across languages.

## Conclusions

This study presents a large-scale set of subjective AoA norms for 30,849 Russian words, offering comprehensive and reliable estimates of lexical acquisition age. The dataset demonstrates high internal consistency and strong convergent validity in relation to objective vocabulary-testing measures as well as with previously published Russian AoA data. The expected relations with frequency, word length, concreteness, imageability, and familiarity further support the construct validity of the ratings. By substantially extending the lexical coverage of available Russian psycholinguistic norms, the present resource enables more precise control of lexical variables in experimental studies and facilitates cross-linguistic comparisons of AoA effects. The norms, publicly available on OSF, can serve as a valuable tool for research in psycholinguistics, reading and vocabulary development, computational modeling, and applied language studies.

## Data Availability

The AoA norms for 30,849 Russian words and test materials are available on OSF: https://osf.io/56da9/

## Appendix

### Instruction for the paper-and-pencil format of data collection

**Table:**

*Original instruction (in Russian)*	*English translation*
Вам будут предъявлены слова. Ваша задача - определить примерный возраст, в котором Вы усвоили предъявляемое слово. Усвоить слово – это значит понимать смысл слова. В раннем детстве слова воспринимаются на слух; многие из них мы начинаем понимать раньше, чем произносить. Усвоенным считается не только то слово, которое Вы используете в своей речи, но и слово, смысл которого Вы понимаете, когда слышите (от родителей, друзей, преподавателей и т.п.) или читаете его. Постарайтесь указать именно тот возраст, когда Вы начали понимать смысл этого слова. Например, если Вы считаете, что слово «дракон» Вы усвоили в возрасте трех лет, напишите напротив него цифру "3". Если Вы считаете, что слово «сноб» Вы усвоили в возрасте 16 лет, напишите «16». Если Вы не знаете, что означает какое-то слово, напишите напротив букву «Н». Все слова взяты из современного словаря русского языка. Среди них есть как простые и широко употребляемые слова (от существительных до предлогов и союзов), так и относительно редкие и малоизвестные. Постарайтесь дать наиболее точные оценки возраста усвоения каждого слова, но не думайте слишком долго над одним словом. Не стесняйтесь ставить "Н", когда смысл слова Вам не известен. Это полезнее для конечного результата, чем указание выдуманного возраста усвоения.	You will be presented with words. Your task is to indicate the approximate age at which you acquired each word. To acquire a word means to understand its meaning. In early childhood, words are learned through listening, and we begin to understand many of them before we can pronounce them. A word is considered acquired not only if you use it in your own speech, but also if you understand its meaning when you hear it from others, such as parents, friends, or teachers, or when you read it. Please indicate the age at which you began to understand the meaning of each word. For example, if you think you acquired the word “dragon” at the age of three, write “3” next to it. If you think you acquired the word “snob” at the age of 16, write “16.” If you do not know the meaning of a word, write the letter “Н” next to it. All words are taken from a modern dictionary of Russian. They include both simple and widely used words, ranging from nouns to prepositions and conjunctions, and relatively rare and unfamiliar ones. Please provide the most accurate estimate you can of the age at which you acquired each word, but do not spend too much time on any one word. Do not hesitate to write “Н” when you do not know the meaning of a word. This is more helpful for the final results than giving a made-up age of acquisition.

### Calibrator words

**Table:**

Lists 1-25		Lists 26-100		Lists 101-103	
*Calibrator word*	*English translation*	*Calibrator word*	*English translation*	*Calibrator word*	*English translation*
рука	arm; hand	пить	to drink	спать	to sleep (imperf.)
муравей	ant	счастливый	happy; lucky	счастливый	happy; lucky
пить	to drink	грядка	garden bed	грядка	garden bed
галстук	tie, necktie	идти	to go, to walk (imperf.)	идти	to go, to walk (imperf.)
ломать	to break (imperf.)	зелёный	green	синий	Blue
сигара	cigar	политика	politics	политика	politics
разжигать	to kindle (imperf.)	ранний	early	ранний	Early
зелёный	green	превзойти	to surpass (perf.)	превзойти	to surpass (perf.)
идеалистический	idealistic	аксиома	axiom	аксиома	Axiom
дирижировать	to conduct	похудеть	to lose weight (perf.)	похудеть	to lose weight (perf.)

## Footnotes

1 We are deeply grateful to Marina Kolbeneva and Yuri Alexandrov, who provided us with their data in a form convenient for analysis.

2 https://github.com/LuminosoInsight/exquisite-corpus
